# Impact of Prolonged Blood Incubation and Extended Serum Storage at Room Temperature on the Human Serum Metabolome

**DOI:** 10.3390/metabo8010006

**Published:** 2018-01-13

**Authors:** Beate Kamlage, Sebastian Neuber, Bianca Bethan, Sandra González Maldonado, Antje Wagner-Golbs, Erik Peter, Oliver Schmitz, Philipp Schatz

**Affiliations:** 1 Metanomics Health GmbH, Tegeler Weg 33, 10589 Berlin, Germany; sebastian.neuber@metanomics-health.de (S.N.); bianca.bethan@metanomics-health.de (B.B.); antje.wagner-golbs@metanomics-health.de (A.W.-G.); jerikpeter@googlemail.com (E.P.); 2 Metanomics GmbH, Tegeler Weg 33, 10589 Berlin, Germany; sandra.gonzalez@outlook.com (S.G.M.); oliver.schmitz@metanomics.de (O.S.); 3 Precision Medicine Unit, Precision Medicine and Genomics, IMED Biotech Unit, AstraZeneca, 43183 Mölndal, Gothenburg, Sweden; philipp.schatz@astrazeneca.com

**Keywords:** biomarker, pre-analytical phase, serum, metabolomics, quality control, biobanking, mass spectrometry

## Abstract

Metabolomics is a powerful technology with broad applications in life science that, like other -omics approaches, requires high-quality samples to achieve reliable results and ensure reproducibility. Therefore, along with quality assurance, methods to assess sample quality regarding pre-analytical confounders are urgently needed. In this study, we analyzed the response of the human serum metabolome to pre-analytical variations comprising prolonged blood incubation and extended serum storage at room temperature by using gas chromatography-mass spectrometry (GC-MS) and liquid chromatography-tandem mass spectrometry (LC-MS/MS) -based metabolomics. We found that the prolonged incubation of blood results in a statistically significant 20% increase and 4% decrease of 225 tested serum metabolites. Extended serum storage affected 21% of the analyzed metabolites (14% increased, 7% decreased). Amino acids and nucleobases showed the highest percentage of changed metabolites in both confounding conditions, whereas lipids were remarkably stable. Interestingly, the amounts of taurine and *O*-phosphoethanolamine, which have both been discussed as biomarkers for various diseases, were 1.8- and 2.9-fold increased after 6 h of blood incubation. Since we found that both are more stable in ethylenediaminetetraacetic acid (EDTA) blood, EDTA plasma should be the preferred metabolomics matrix.

## 1. Introduction

Metabolomics on human blood-based samples, such as serum or plasma, can complement proteomics and transcriptomics as a physiological “downstream result” of the -omics cascade, and has considerable potential in clinical diagnostics and pharmaceutical research [1]. The discovery of novel biomarkers for early diagnosis, prognosis and prediction of various diseases, the identification of new drug targets, the characterization of drug adverse effects, and the monitoring of treatment response are just a few examples where metabolomics could be applied to improve medical care and support precision medicine strategies. Though blood-based samples are particularly attractive in these research fields because of their minimally invasive accessibility and the comprehensive coverage of the human metabolic landscape, the effects of variations in sample processing must be properly addressed when analyzing serum and plasma metabolomes [2,3,4,5,6,7,8,9,10,11,12]. The potential failure of metabolic biomarker research due to pre-analytical reasons was reviewed [13], and pre-analytical challenges were observed in transcriptomics, proteomics, and peptidomics studies [14,15,16,17]. Therefore, high-quality biospecimens and an improved quality assurance with standard operating procedures (SOPs) are important to obtain reliable and reproducible results from metabolomics and other -omics studies [18,19,20,21,22,23,24]. However, this may not always be feasible. For example, the analysis of samples is less likely in biobanks where the SOPs are not always as detailed as necessary, or not well documented. In these scenarios, quality management would greatly benefit from quality control measures applicable to existing samples for assessing their pre-analytical quality. 

We have recently shown that delays in blood and plasma processing at room temperature (RT) have an enormous impact on the human EDTA plasma metabolome [9]. Since serum samples represent one of the most common matrices used in clinical routine and stored in biobanks [25], in this study, we have analyzed the effect of clinically relevant confounders on the human serum metabolome. The aim of our work was to analyze the impact of prolonged blood incubation and extended serum storage at RT on the human serum metabolome using gas chromatography-mass spectrometry (GC-MS) and liquid chromatography-tandem mass spectrometry (LC-MS/MS)-based methods.

## 2. Results

### 2.1. Discrimination of Pre-Analytical Confounded and Control Sample Groups 

**Figure 1:**
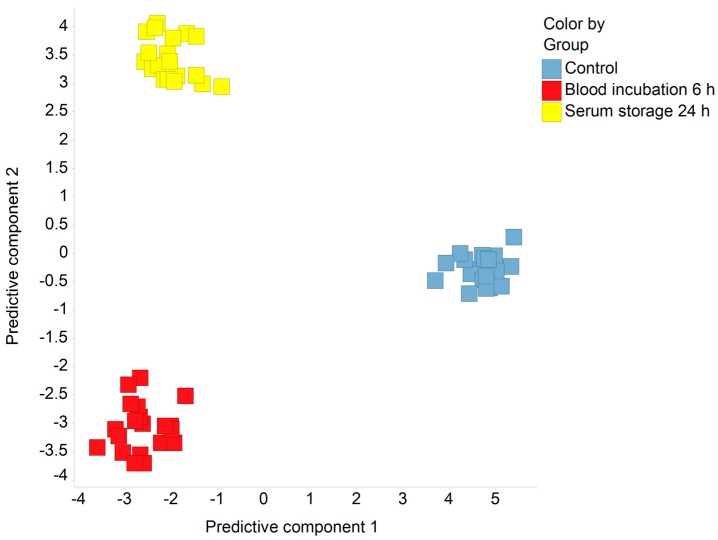
Orthogonal projection to latent structures-discriminant analysis (OPLS-DA) on metabolomics data. The scores of the first and the second predictive components were calculated from log10-transformed metabolomics data. Q^2^_cum_ = 0.87.

After performing a MS-based metabolomics broad profiling approach, we conducted a supervised orthogonal projection to latent structures-discriminant analysis (OPLS-DA) to get a first overview on the data set (Figure 1). The resulting plot revealed a clear clustering of the different sample processing conditions (control, blood incubation for 6 h, and serum storage for 24 h) with a cumulative Q^2^ value of 0.87. This clustering behavior was not observed in an unsupervised principal component analysis (not shown), indicating that inter-individual variability and sex have a greater impact on the serum metabolome than the applied confounders. 

### 2.2. Pre-Analytical Variation Affects the Serum Metabolome

Based on the acquired metabolomics data, we investigated the effects of pre-analytical variation during blood and serum processing on the human serum metabolome (Table 1). We found that prolonged incubation of blood at RT (6 h versus 30 min) affected 24% of 225 tested serum metabolites (20% increased, 4% decreased), and extended storage of serum at RT (24 h) resulted in a statistically significant increase of 14%, and a decrease of 7%, of the analyzed metabolites. Amino acids and nucleobases showed the highest percentage (up to 82%) of changed metabolites in both prolonged blood incubation and extended serum storage. Further, 73% of energy metabolism-related metabolites and 60% of carbohydrates were significantly changed by prolonged blood incubation, but were less sensitive to extended serum storage. In contrast, lipids were highly stable towards both applied confounders. Details of statistical analysis are given in Tables S1 and S2 in the Supplement.

**Figure 2:**
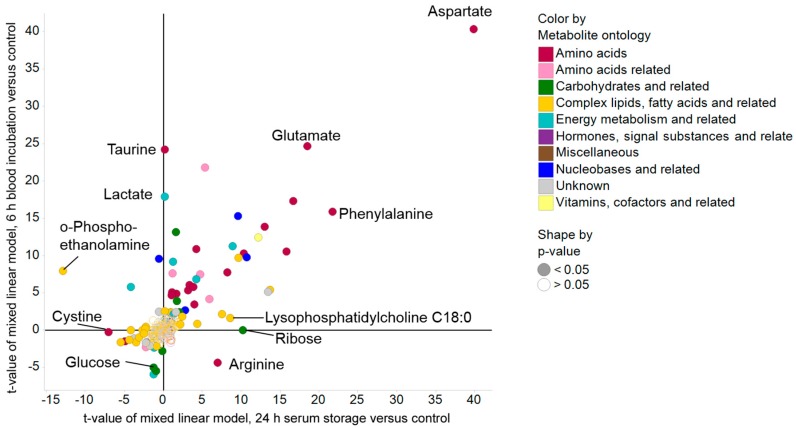
Scatter plot of *t*-values using ANOVA to visualize effects of prolonged blood incubation and extended serum storage versus control. The magnitude of *t*-values is indicative for the difference in metabolite values relative to their variance, and as such, a high absolute *t*-value represents a more robust change.

To assess the magnitude of the changes in metabolite levels occurring in response to the two applied pre-analytical confounders, the *t*-values of mixed linear models using the control group as reference were plotted (Figure 2). Generally, *t*-values represent the fold change in units of standard deviations, and are positive for increases and negative for decreases relative to the control group. Therefore, a higher magnitude of *t*-values not only contains information about the observed difference between two groups, such as the absolute change in score, it also has information regarding the variability of the respective metabolite. In this context, we found that amino acids showed the highest magnitude of changes after pre-analytical variation with a significant increase after both prolonged blood incubation and extended serum storage at RT, indicating continuous activity of proteases and/or peptidases during blood and serum processing. The only amino acid that showed markedly decreased *t*-values after prolonged blood incubation was arginine, which was probably due to the arginase activity of erythrocytes [26]. Complex lipids, fatty acids, and related metabolites were more robust towards the applied confounders (Table S2), except for lysophosphatidylcholines, which were increased upon extended serum storage at RT (Table S1), likely because of phospholipase A activity [27]. 

### 2.3. Impact of Pre-Analytical Variation on Selected Serum Metabolites

**Figure 3:**
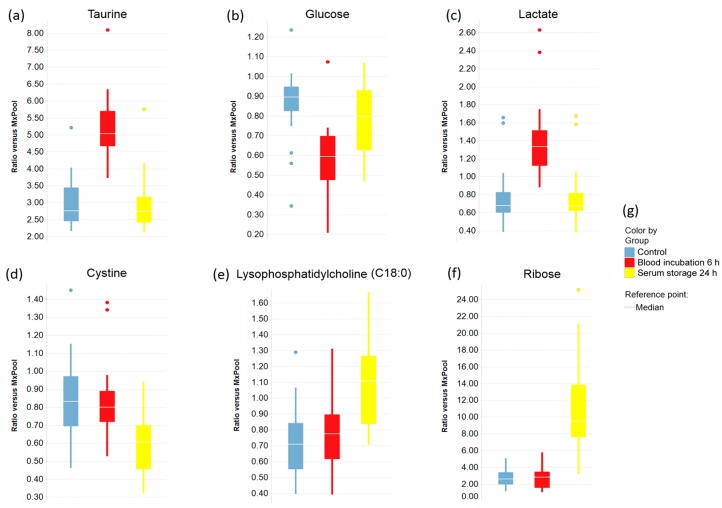
The effects of prolonged blood incubation and extended serum storage on selected metabolites are represented by boxplots. (**a**–**c**) Metabolite levels that were significantly changed by prolonged blood incubation; (**d**–**f**) Metabolite amounts that were significantly changed by extended serum storage; (**g**) Legend for the boxplots in (**a**–**f**).

**Figure 4:**
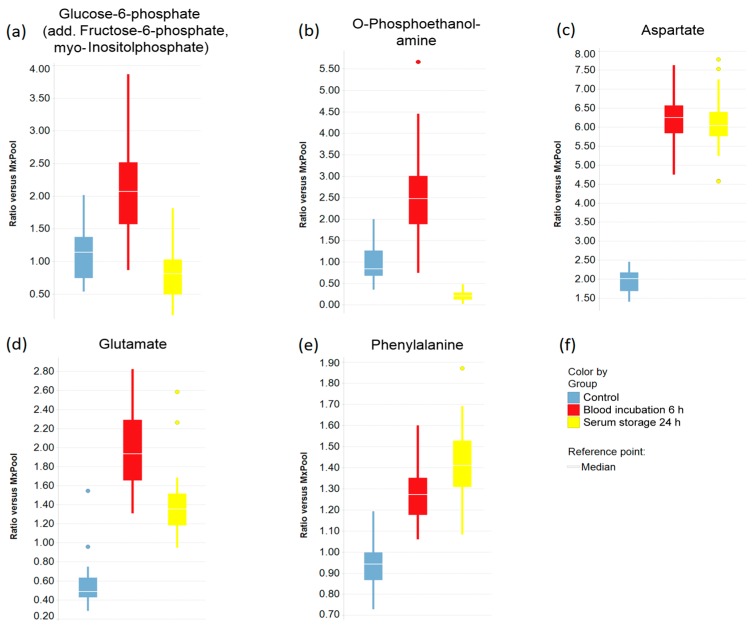
Selected metabolites that were affected by both prolonged blood incubation and extended serum storage. (**a**–**e**) Boxplots of metabolite levels that were significantly changed by both prolonged blood incubation and extended serum storage; In (**a**), the term “add.” (additional) means that quantification could be influenced by minor levels of other metabolites with identical analytical characteristics with respect to the quantitation method; (**f**) Legend for the boxplots in (**a**–**e**).

**Figure 5:**
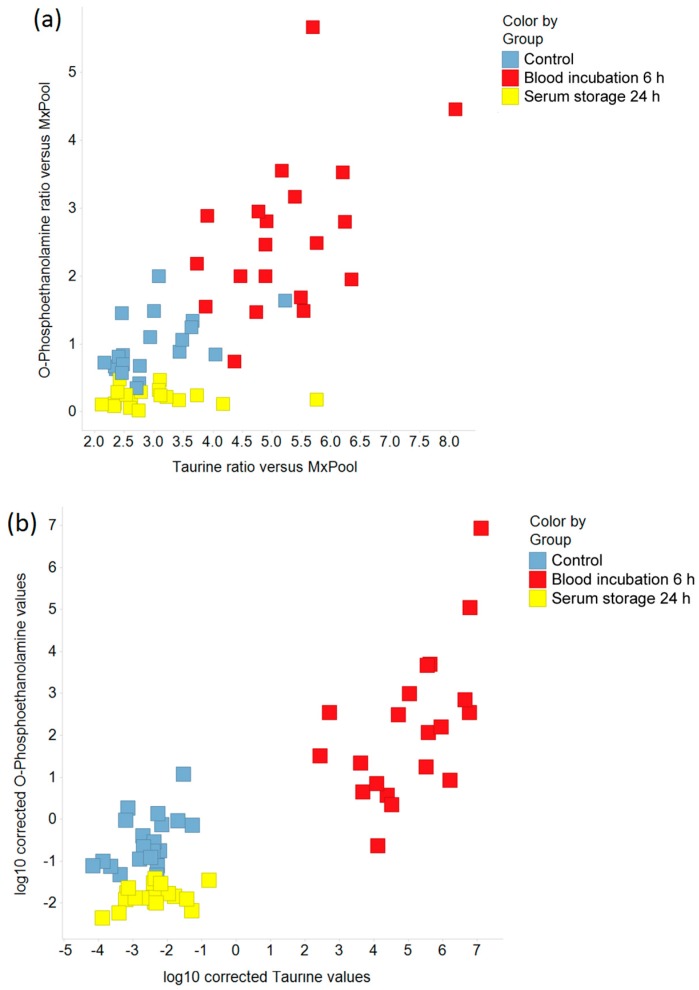
The effects of prolonged blood incubation and extended serum storage on taurine and *O*-phosphoethanolamine are shown by scatter plots. (**a**) Ratios of taurine and *O*-phosphoethanolamine relative to the MxPool; (**b**) Logarithmic (base 10) values for taurine and *O*-phosphoethanolamine were corrected for subject and sex to account for inter-individual variability.

Boxplots were created to indicate the effects of prolonged blood incubation and extended serum storage at RT on selected metabolites (Figure 3 and Figure 4). Taurine, glucose, and lactate (Figure 3a–c) were all greatly affected by prolonged blood incubation for 6 h; the amounts of taurine and lactate were increased, whereas the glucose level was decreased. Figure 3d–f shows boxplots of cystine, lysophosphatidylcholine (C18:0), and ribose, which were all impacted by extended serum storage for 24 h; the cystine amount was decreased while lysophosphatidylcholine (C18:0) and ribose levels were strongly increased. Figure 4 displays boxplots of glucose-6-phosphate (add. fructose-6-phosphate, myo-inositol-phosphate), *O*-phosphoethanolamine, and the amino acids aspartate, glutamate, and phenylalanine, which were all affected by both a 6 h-blood incubation and a 24 h-serum storage at RT. Interestingly, the level of *O*-phosphoethanolamine was 2.7-fold increased upon prolonged blood incubation, and showed an 80% decrease upon extended serum storage. The scatter plots of taurine and *O*-phosphoethanolamine (Figure 5a) revealed a separation of samples based on their pre-analytical variation. Furthermore, this discriminatory effect of both metabolites was enhanced when ratios were corrected for subject and sex (Figure 5b). Given this background, detailed SOPs for sample processing are necessary in order to successfully develop taurine and *O*-phosphoethanolamine as commercial biomarkers in serum.

### 2.4. Impact of Pre-Analytical Variation on Taurine and O-Phosphoethanolamine in EDTA Blood and Plasma Samples

**Figure 6:**
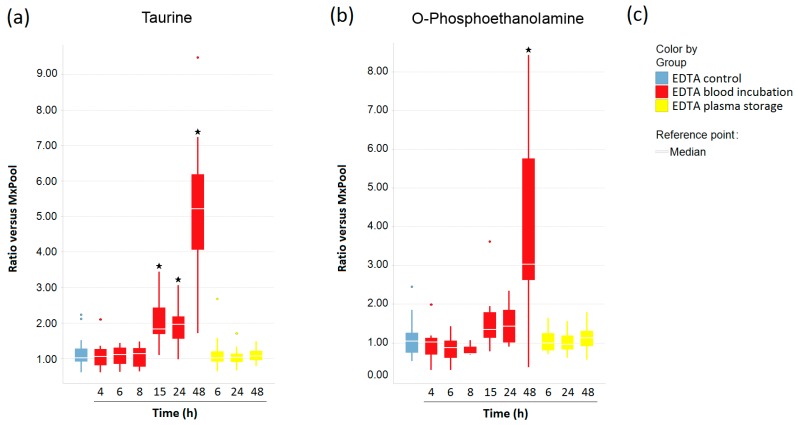
The impact of pre-analytical variations on (**a**) taurine and (**b**) *O*-phosphoethanolamine concentrations in EDTA blood and plasma, represented by boxplots; In (**a**,**b**), stars indicate significant differences compared with the control group, calculated by using ANOVA with a significance level of *p* < 0.05; (**c**) Legend for the boxplots in (**a**,**b**).

After having shown that the amounts of taurine and O-phosphoethanolamine were increased after a 6 h-blood incubation at RT post-blood draw (Figure 3a and Figure 4b), we next asked whether there is a similar effect on these metabolites in EDTA blood. As depicted in Figure 6a, taurine levels did not change after prolonged EDTA blood incubation for 4 h to 8 h. Only by applying blood incubation periods of more than 15 h were taurine concentrations significantly increased. Similarly, O-phosphoethanolamine was relatively stable when EDTA blood was processed within 4 h to 24 h post-blood draw, but was threefold increased after a 48 h-blood incubation (Figure 6b). In contrast to this, extended storage of EDTA plasma of up to 48 h at RT neither affected taurine nor *O*-phosphoethanolamine levels (Figure 6a,b). 

## 3. Discussion

The high sensitivity of modern analytical techniques coupled with the minimally invasive accessibility of human plasma, serum, or urine samples, and the responsiveness of the metabolome to pathological alterations, make metabolomics an attractive approach for multiple biomedical applications. However, since metabolites are also sensitive to pre-analytical confounders, accurate quality control of the pre-analytical phase is mandatory for metabolite profiling. The results of this short-term stability study demonstrated that both prolonged incubation of blood and extended storage of serum at RT affect the human serum metabolome. Amino acids and nucleobases showed the highest percentage of changed metabolites, whereas lipids were generally more stable towards both applied confounders, with lysophosphatidylcholines being the exception.

By using supervised and unsupervised multivariate data analyses, we found that the two main aspects of human serum metabolome variability were inter-individual variability and sex. This fact was described before by Hirayama et al. [10] using serum and plasma samples from only four volunteers, and has now been confirmed by using a larger number of individuals. Moreover, the supervised OPLS-DA model revealed that the impact of pre-analytical variation on selected serum metabolites is greater than that of inter-individual variability and sex. Hence, several metabolites were changed significantly upon prolonged blood incubation or extended serum storage relative to control samples. As one example, the amount of *O*-phosphoethanolamine was 2.7-fold increased after a 6-h incubation of blood, which can be interpreted by platelet-released sphingosine-1-phosphate in the sphingolipid signaling pathway, generating *O*-phosphoethanolamine by sphingosine-1-phosphate lyase activity [28] [EC 4.1.2.27]. Upon extended serum storage (24 h), the amount of *O*-phosphoethanolamine was reduced by 80% compared with control samples, probably due to degradation by phosphoethanolamine/phosphocholine phosphatase activity [EC 3.1.3.75]. Since *O*-phosphoethanolamine was reported to inhibit mitochondrial respiratory activity [29], and thus represents a promising biomarker for associated diseases, our findings regarding sample processing should be considered for future development of biomarker panels containing *O*-phosphoethanolamine. Given this background, detailed SOPs for sample processing are necessary to achieve high-quality serum samples. 

An interesting finding of our study was that taurine levels were significantly increased due to prolonged blood incubation at RT. Taurine is a highly abundant amino acid in mammals, and can be found in blood plasma and blood cells. It is essential for cats and conditionally essential for primates, and has a role in the development of central nervous, renal, cardiovascular, reproductive, and immune systems [30]. Circulating taurine was reported to be particularly accumulated in leukocytes and platelets [31,32]. Interestingly, systemic taurine supplementation has been shown to stabilize platelets against aggregation-inducing stimuli [33]. However, since taurine is synthesized and released by platelets [34,35], the increased taurine values observed upon prolonged blood incubation at RT are probably the result of platelet metabolism. In our previous study analyzing the impact of pre-analytical variation on EDTA plasma, taurine was significantly decreased upon the prolonged storage of EDTA blood at 4 °C [9]. This difference might be explained by temperature-induced platelet shape changes during EDTA blood incubation, and the consequently improved sedimentation behavior during centrifugation and fewer remaining platelets in the derived plasma from which taurine is released during sample freezing/defrosting or during sample preparation for analysis. Previous studies based on serum samples indicated that taurine is a potential biomarker for preeclampsia [36]. However, in these experiments, blood was centrifuged within 6 h after blood draw. Our results presented here showed that taurine increased about 1.8-fold when blood samples were centrifuged after 6 h post-blood draw compared with 30 min of coagulation. Therefore, it is mandatory to implement quality control measures for biomarker-related research to achieve reliable results and ensure reproducibility. 

Another important result of this work is that taurine and *O*-phosphoethanolamine were more stable during prolonged blood incubation when EDTA was added to the blood samples. Moreover, the fact that pre-analytical variation mainly affected amino acids is a finding that is different from our previously published EDTA plasma study [9]. EDTA has a stabilizing effect on metabolites due to chelation of divalent cations, which are needed for several enzymatic activities. Therefore, it may be concluded that EDTA plasma is better suited for metabolomics than serum. Nevertheless, high-quality serum samples collected and processed according to strictly defined SOPs will be suitable as well. In this context, serum samples are especially interesting, because amino acid concentrations are generally found to be higher in serum than in plasma [6,37,38]. 

The finding that lysophosphatidylcholine (C18:0) levels were increased upon extended serum storage may be explained by phospholipase A2 activity [27]. Increased amounts of amino acids caused by extended serum storage were obtained due to serum proteinase activity, and augmented glucose-6-phosphate concentrations that were obtained because of prolonged blood incubation most probably originated from the pentose phosphate cycle in erythrocytes. The observation that prolonged blood incubation leads to increased lactate concentrations and decreased glucose levels is already known and was thus confirmed, and can be explained by anaerobic glycolysis of erythrocytes [39].

The knowledge of pre-analytical factors that affect serum and plasma sample quality is essential for understanding underlying mechanisms and controlling their impact. Therefore, sample quality must be proven, and SOPs for sample processing as well as shipment logistics should be revised. The implementation of tools to assess the quality of existing samples could be especially important for samples from biobanks where detailed information on sample processing are not always available. Limitations of our studies include the low number (20) of subjects, the inclusion of healthy subjects only, the customized study design, and the relatively small number of confounders. In further studies, preferably on clinical samples from biobanks, one should also consider: samples from patients with various diseases, further confounding factors such as different centrifugation conditions, hemolytic, icteric and lipemic samples, blood incubation periods < 6 h, and serum storage times < 24 h at RT.

In sum, the human serum metabolome is sensitive to prolonged blood incubation (6 h) and extended serum storage (24 h) at RT. Thus, in clinical research, and particularly in -omics studies, it is mandatory to establish a stringent quality management for sample processing to achieve reliable results and ensure reproducibility.

## 4. Material and Methods

### 4.1. Serum and EDTA Plasma Samples

The study was conducted in adherence to the Declaration of Helsinki, and protocols were approved by the local ethics committee. All of the subjects gave their informed consent before they participated in the study. The whole process from blood draw to sample freezing was performed by in.vent Diagnostica GmbH (Hennigsdorf, Germany). All blood, plasma, and serum processing steps and centrifugation procedures were done at RT. 

Human serum samples were collected from self-reported healthy volunteers who were randomly chosen from a database of 200 volunteers. Twenty subjects, 15 female and five male individuals with a median age of 37 years and a median body mass index of 24 kg/m^2^ were recruited. Inclusion criteria were 18 to 60 years of age, a body mass index of 17.5 to 30.5 kg/m^2^, and 8 h to 12 h fasting before blood draw. Exclusion criteria were anemia, pregnancy (second or third trimester), and acute infections. Blood was taken in the morning after overnight fasting using a 20-gauge safety-fly blood collection system. Therefore, the volunteers were kept in an upright sitting position, and the tourniquet was released after blood flow started. If the peripheral venipuncture was unsuccessful on the first attempt, the procedure was repeated once on the other arm. After discarding the first 1 mL of blood to avoid potential contamination with skin epithelial cells, approximately 15 mL of blood was collected in two serum S-Monovette^®^ tubes (9 mL and 4.9 mL). The blood in the 9-mL serum S-Monovette coagulated for 30 min, followed by centrifugation at 2000× *g* for 10 min (manufacturer’s recommended centrifugation condition). Then, the resulting serum supernatant was transferred to a new tube. The sample was mixed by inverting several times and divided into two portions. One aliquot was immediately frozen in 0.5 mL-portions at −80 °C (group “control”), while the other was incubated for 24 h before being stored in 0.5 mL-portions at −80 °C (group “serum storage 24 h”). The 4.9 mL-blood sample was incubated for 6 h followed by centrifugation as described above, and the resulting serum supernatant was stored in 0.5 mL-aliquots at –80 °C (group “blood incubation 6 h”). The pre-analytical confounders (“blood incubation 6 h” and “serum storage 24 h”) were chosen based on previously published representative papers [40,41] and criteria formulated by biobank experts to mimic delays in sample processing that often occur in clinical practice. 

Human EDTA plasma samples were collected altogether from 20 self-reported healthy individuals. Inclusion criteria were 18 to 60 years of age, a body mass index of 17.5 to 30.5 kg/m^2^, and 8 h to 12 h fasting before blood draw. Exclusion criteria were the presence of acute or chronic diseases, anemia, and pregnancy (second or third trimester). Blood was taken as described above, and collected in two 9 mL-EDTA S-Monovette^®^ tubes. Nine mL of blood was centrifuged according to the instructions of the manufacturer, and the resulting plasma supernatant was divided into four portions. One aliquot was immediately frozen in 0.5 mL-portions at −80 °C (group “EDTA control”), while the others were incubated for 6 h, 24 h, and 48 h, respectively, before being stored in 0.5 mL-portions at −80 °C (group “EDTA plasma storage”). Another 9 mL of blood was divided into six aliquots and incubated for either 4 h, 6 h, 8 h, 15 h, 24 h or 48 h, followed by centrifugation according to the manufacturer’s instructions. The resulting plasma supernatants were immediately frozen in 0.5 mL-portions at −80 °C (group “EDTA blood incubation”). Within each confounder group, samples were collected from 10 out of the 20 subjects. 

Frozen samples were shipped on dry ice to our analytical laboratory, and stored at −80 °C until use.

### 4.2. Metabolite Profiling Analysis

Two types of MS analyses were applied to serum and EDTA plasma samples: GC-MS (Agilent 6890 GC connected to an Agilent 5973 MS system) and LC-MS/MS (Agilent 1100 high-performance liquid chromatography system coupled to an Applied Biosystems API4000 MS/MS System) were used for MxP^®^ Broad Profiling, as described elsewhere [42,43,44]. In brief, proteins were removed from samples by precipitation using three volumes of acetonitrile. Subsequently, polar and non-polar fractions were separated by adding water and an ethanol/dichloromethane solution (2:1). For LC-MS/MS analysis, both fractions were reconstituted in appropriate solvent mixtures, and high-performance liquid chromatography was performed by gradient elution using methanol/water/formic acid on reversed phase separation columns. For GC-MS analysis, the non-polar fraction was treated with methanol under acidic conditions to give fatty acid methyl esters that were derived from both free fatty acids and hydrolyzed complex lipids. The polar and non-polar fractions were further derivatized with *O*-methyl-hydroxylamine hydrochloride to convert oxo-groups to *O*-methyloximes, and subsequently with a silylating agent (*N*-methyl-*N*-(trimethylsilyl) trifluoroacetamide) before analysis. The mass spectrometric detection technology was applied as described in patent WO2003073464 [45], which allowed for targeted and high-sensitivity multiple reaction monitoring (MRM) profiling in parallel to a full screen analysis. In short, MS detection was performed with repetitive cycles of MRM transitions for pre-selected metabolites followed by a full scan from a mass-to-charge ratio of 100 to 1000. The instrument was operated in negative ionization mode for compounds in the polar fraction, and in positive ionization mode for metabolites in the non-polar fraction. All of the samples were analyzed once in randomized analytical sequence design to avoid artificial results with respect to analytical shifts. Metabolite identification was mainly carried out by comparison to authentic standards. In addition, further metabolites were identified via intensive structure elucidation experiments, such as high-resolution measurements using Fourier transform ion cyclotron resonance MS (Bruker Solarix), the addition of salts, peak purification via fractionation, and MS experiments. These procedures led to further information on metabolite structures, such as atmospheric pressure chemical ionization. Internal standards were added to increase precision, quality control the entire analytical process, and monitor the stability of the measurement. The internal standards used within this study were either isotope-labeled compounds or chemicals representing different chemical structures and polarities such as amino acids, carbohydrates, cofactors, and lipids.

### 4.3. Data Normalization and Nomenclature

To account for inter- and intra-instrumental variation, metabolite concentrations were calculated relative to metabolite levels in reference samples derived from aliquots of all individual samples, resulting in semi-quantitative data. To allow an experiment-to-experiment alignment of data sets, the semi-quantitative data were further normalized to standardized MxPool samples [9]. Both types of reference samples were run in parallel throughout the entire procedure. Details regarding the metabolite nomenclature are available [43], but in brief, the term “additional” (add.), was applied to indicate that quantification can be influenced by metabolites exhibiting identical analytical characteristics with respect to the quantitation method. 

### 4.4. Statistical Analysis and Data Visualization

Prior to statistical analysis, the transformation to base 10 logarithms (log10) was done to approximate a normal distribution of the data. The programs R (versions 2.8.1 and 2.14.1, packages nlme and glmnet), SIMCA (version 14.1) and Tibco Spotfire (version 6.0) were used for data analysis and visualization. Statistical analysis was done via a mixed linear model analysis of variance (ANOVA) with “subject” as the random intercept and “group”, “sex”, “age”, and “body mass index” as fixed effects. Restricted maximum likelihood was used to estimate consistent variance components for the linear mixed-effects models [46]. Significance level was set to 5%. The multiple test problem was addressed by calculating the false discovery rate (FDR) using the Benjamini–Hochberg procedure [47]. In addition, multivariate analysis was performed by OPLS-DA [48,49], which is a commonly used method for discovering relationships between metabolomics data and different sample groups such as pre-analytical confounders or controls. To ensure validation of the OPLS-DA model, the cross-validated cumulative Q^2^ value was used as a measure for the predictive value of this approach. 

## Figures and Tables

**Table 1:** Significant metabolite changes by applying prolonged blood incubation or extended serum storage. Statistical analysis was done via analysis of variance (ANOVA), the significance level was set to *p* < 0.05, and false discovery rate (FDR) < 0.2. Further details are listed in Tables S1 and S2.

Group	Metabolite Ontology Class (Number)	Significantly Changed Metabolites versus the Control Group (Increase/Decrease)	
		Number	Percent Change
Blood incubation (6 h)	All (225)	54 (45/9)	24 (20/4)
	Amino acids (22)	18 (17/1)	82 (77/5)
	Amino acids related (15)	5 (4/1)	33 (27/7)
	Carbohydrates and related (10)	6 (3/3)	60 (30/30)
	Complex lipids, fatty acids and related (99)	8 (7/1)	8 (7/1)
	Energy metabolism and related (11)	8 (6/2)	73 (55/18)
	Hormones (2)	0 (0/0)	0 (0/0)
	Miscellaneous (9)	0 (0/0)	0 (0/0)
	Nucleobases and related (5)	4 (4/0)	80 (80/0)
	Vitamins, cofactors, and related (6)	1 (1/0)	17 (17/0)
	Unknowns (46)	4 (3/1)	9 (7/2)
Serum storage (24 h)	All (225)	48 (32/16)	21 (14/7)
	Amino acids (22)	16 (14/2)	73 (64/9)
	Amino acids related (15)	4 (3/1)	27 (20/7)
	Carbohydrates and related (10)	1 (1/0)	10 (10/0)
	Complex lipids, fatty acids, and related (99)	17 (7/10)	17 (7/10)
	Energy metabolism and related (11)	3 (2/1)	27 (18/9)
	Hormones (2)	0 (0/0)	0 (0/0)
	Miscellaneous (9)	0 (0/0)	0 (0/0)
	Nucleobases and related (5)	3 (3/0)	60 (60/0)
	Vitamins, cofactors, and related (6)	1 (1/0)	17 (17/0)
	Unknowns (46)	3 (1/2)	7 (2/4)

## Supplementary Materials

The following are available online at www.mdpi.com/2218-1819/8/1/6/s1. Table S1: Impact of pre-analytical confounders on human serum metabolite concentrations, Table S2: Metabolites in human serum that were found to be robust to pre-analytical confounders.
