# Supplementary material for: Impact of Prolonged Blood Incubation and Extended Serum Storage at Room Temperature on the Human Serum Metabolome
Source: Metabolites. 2018 Jan 13;8(1):6. doi: 10.3390/metabo8010006 (PMC5875996; doi:10.3390/metabo8010006)
Supplement: Supplementary file 1 [file metabolites-08-00006-s001.pdf]

Table S1: Impact of preanalytical confounders on human serum metabolite concentrations. Six analytes with final chemical structure identity pending are not given. Statistical analysis was done via ANOVA models (see Material and Methods). A p-value of < 0.05 and a FDR of < 0.2 was considered as statistically significant.

Ontology 1	Ontology 2	Metabolite	Serum storage 24 h, ratio versus control	Blood incubation 6 h, ratio versus control	Serum storage 24 h, p-value versus control	Blood incubation 6 h, p-value versus control	Serum storage 24 h, t-value versus control	Blood incubation 6 h, t-value versus control	Serum storage 24 h, FDR	Blood incubation 6 h, FDR	Sex, ratio male versus female	Sex, p-value male versus female	Sex, t-value male versus female
Amino acids	Amino acids, acidic	Aspartate	3.16	3.21	1.31E-32	8.24E-33	39.85	40.35	2.96E-30	1.85E-30	1.08	2.82E-01	1.11
	Amino acids, acidic	Glutamate	2.68	3.72	1.29E-20	5.17E-25	18.52	24.70	9.69E-19	5.82E-23	1.33	5.68E-03	3.19
	Amino acids, aromatic	Phenylalanine	1.52	1.36	4.91E-23	2.27E-18	21.73	15.89	5.53E-21	7.31E-17	1.10	1.31E-01	1.59
	Amino acids, aromatic	Tyrosine	1.06	1.05	2.76E-04	1.34E-03	4.02	3.47	1.88E-03	7.56E-03	1.02	8.17E-01	0.24
	Amino acids, basic	Arginine	1.28	0.86	3.23E-08	9.82E-05	6.96	-4.37	3.47E-07	5.97E-04	1.10	4.29E-01	0.81
	Amino acids, basic	Asparagine	1.04	1.19	2.97E-01	3.25E-05	1.06	4.71	6.33E-01	2.03E-04	1.02	8.42E-01	0.20
	Amino acids, basic	Histidine	1.09	1.14	4.46E-04	9.88E-07	3.85	5.83	2.95E-03	8.24E-06	1.06	6.78E-01	0.42
	Amino acids, basic	Lysine	1.05	1.14	1.40E-04	3.02E-13	4.23	10.89	1.05E-03	5.23E-12	0.97	7.58E-01	-0.31
	Amino acids, branched chain	Isoleucine	1.02	1.07	1.02E-01	1.80E-05	1.67	4.90	3.66E-01	1.16E-04	1.39	4.01E-05	5.60
	Amino acids, branched chain	Leucine	1.16	1.17	1.46E-15	1.91E-16	13.00	13.86	4.12E-14	4.78E-15	1.33	2.09E-04	4.77

	Amino acids, branched chain	Valine	1.07	1.06	5.34E-10	2.19E-09	8.25	7.78	6.68E-09	2.35E-08	1.25	7.52E-03	3.06
	Amino acids, neutral	Alanine	1.11	1.11	2.39E-12	2.73E-12	10.36	10.31	4.22E-11	4.10E-11	1.06	5.43E-01	0.62
	Amino acids, neutral	Glycine	1.30	1.19	2.53E-18	7.37E-13	15.84	10.56	1.14E-16	1.18E-11	1.00	9.89E-01	-0.01
	Amino acids, neutral	Proline	1.01	1.05	2.69E-01	1.01E-05	1.12	5.09	6.11E-01	6.89E-05	1.22	1.39E-01	1.56
	Amino acids, neutral	Serine	1.31	1.33	4.39E-19	1.30E-19	16.69	17.30	2.47E-17	4.89E-18	0.97	6.78E-01	-0.42
	Amino acids, neutral	Threonine	1.04	1.07	2.82E-03	4.14E-06	3.19	5.37	1.67E-02	3.01E-05	0.90	5.26E-01	-0.65
	Amino acids, S-containing	Cysteine (add. Cystine)	0.64	0.88	1.60E-05	1.47E-01	-4.94	-1.48	1.44E-04	4.41E-01	1.12	2.53E-01	1.19
	Amino acids, S-containing	Cystine	0.68	0.99	2.18E-08	8.19E-01	-7.04	-0.23	2.45E-07	9.59E-01	1.15	2.76E-01	1.13
	Amino acids, S-containing	Methionine	1.05	1.10	1.75E-03	4.93E-07	3.37	6.05	1.06E-02	4.44E-06	1.16	5.53E-02	2.07
	Amino acids, S-containing	Taurine	1.00	1.76	8.59E-01	2.80E-24	0.18	24.20	9.52E-01	2.10E-22	1.05	7.23E-01	0.36
Amino acids related	Amino acid metabolites	5-Oxoproline (3 additionals)	1.11	1.18	3.27E-05	5.46E-09	4.71	7.49	2.83E-04	5.34E-08	1.17	1.31E-02	2.79
	Amino acid metabolites	beta-Alanine (add. Pantothenic acid)	1.04	1.26	2.33E-01	3.19E-09	1.21	7.66	5.89E-01	3.26E-08	1.44	4.29E-03	3.32
	Collagen metabolism	trans-4-Hydroxyproline	1.13	1.09	7.95E-07	1.72E-04	5.89	4.17	8.13E-06	1.02E-03	1.56	1.28E-01	1.60
	Urea cycle and related	Ornithine	1.11	1.56	4.32E-06	4.48E-23	5.36	21.79	4.05E-05	2.52E-21	1.22	5.57E-02	2.06
	Urea cycle and related	Urea	0.86	0.86	3.17E-02	3.33E-02	-2.27	-2.24	1.55E-01	1.50E-01	1.05	7.45E-01	0.33
Carbohydrates and related	Aminosugars	Glucosamine	0.99	0.86	9.19E-01	8.92E-03	-0.10	-2.76	9.62E-01	4.90E-02	1.04	6.68E-01	0.44

	Monosaccharides	Glucose	0.90	0.67	2.18E-01	1.46E-05	-1.25	-4.97	5.89E-01	9.65E-05	1.02	8.96E-01	0.13
	Monosaccharides	Mannose	0.95	0.76	3.58E-01	3.27E-06	-0.93	-5.45	6.50E-01	2.54E-05	1.05	7.59E-01	0.31
	Nucleobase related saccharides	Ribose	3.79	1.00	2.44E-12	9.87E-01	10.23	0.02	4.22E-11	9.95E-01	1.34	1.37E-01	1.57
	Polyols	myo-Inositol	1.03	1.24	1.15E-01	9.55E-16	1.61	13.18	3.81E-01	2.15E-14	1.08	4.52E-01	0.77
	Polyols	scyllo-Inositol	1.06	1.15	9.41E-02	3.67E-04	1.72	3.91	3.53E-01	2.12E-03	0.99	9.69E-01	-0.04
	Sugar acids	Erythronic acid	1.10	1.12	4.56E-02	1.93E-02	2.07	2.44	2.05E-01	9.44E-02	0.99	9.50E-01	-0.06
Complex lipids, fatty acids and related	Bile acids	Glycochenodeoxycholic acid (add. Glycodeoxycholic acid)	0.91	0.81	3.94E-01	4.19E-02	-0.86	-2.11	6.64E-01	1.78E-01	1.05	9.34E-01	0.08
	Ceramides	Ceramide (d18:1,C24:1) (add. Ceramide (d18:2,C24:0))	1.12	1.04	3.72E-02	4.11E-01	2.16	0.83	1.74E-01	7.57E-01	0.98	9.47E-01	-0.07
	Cholesterylesters	Cholesterylester, total	0.91	0.97	3.80E-03	3.36E-01	-3.08	-0.97	2.19E-02	6.71E-01	0.89	6.24E-01	-0.50
	Fatty acids, mono-unsaturated	Eicosaenoic acid (C20:cis[11]1)	1.04	1.12	3.70E-01	1.92E-02	0.91	2.45	6.50E-01	9.44E-02	1.06	8.05E-01	0.25
	Fatty acids, saturated	Isopalmitic acid (C16:0)	1.04	1.09	2.95E-01	2.56E-02	1.06	2.33	6.33E-01	1.20E-01	0.56	5.80E-02	-2.04
	Lipid hydroperoxides	Phosphatidylcholine hydroperoxide (C16:0,C18:2-OOH)	0.77	0.96	1.82E-02	6.74E-01	-2.51	-0.43	9.74E-02	8.86E-01	0.95	8.31E-01	-0.22
	Lipid precursors	Glycerol-3-phosphate, polar fraction	1.64	1.64	1.20E-11	1.02E-11	9.65	9.71	1.80E-10	1.36E-10	1.03	8.13E-01	0.24
	Lipid precursors	O-Phosphoethanolamine	0.19	2.67	4.76E-15	1.79E-09	-12.88	7.98	1.19E-13	2.01E-08	0.93	7.06E-01	-0.38
	Lysophosphatidylcholines	Lysophosphatidylcholine (C16:0)	0.95	0.98	3.01E-06	1.12E-01	-5.47	-1.63	2.94E-05	3.65E-01	0.98	2.40E-01	-1.22
	Lysophosphatidylcholines	Lysophosphatidylcholine (C17:0)	1.81	1.26	2.75E-16	3.78E-06	13.71	5.40	1.03E-14	2.84E-05	0.89	4.88E-01	-0.71

	Lysophosphatidylcholines	Lysophosphatidylcholine (C18:0)	1.56	1.09	2.10E-10	1.07E-01	8.56	1.65	2.78E-09	3.64E-01	1.02	8.79E-01	0.15
	Lysophosphatidylcholines	Lysophosphatidylcholine (C18:1)	1.22	1.06	4.37E-09	3.42E-02	7.56	2.20	5.18E-08	1.51E-01	1.01	9.01E-01	0.13
	Lysophosphatidylcholines	Lysophosphatidylcholine (C20:4)	1.29	1.05	9.25E-05	3.83E-01	4.37	0.88	7.71E-04	7.48E-01	1.11	4.00E-01	0.86
	Phosphatidylcholines	Phosphatidylcholine (C16:1,C18:2)	0.92	0.96	1.17E-03	1.12E-01	-3.51	-1.63	7.30E-03	3.65E-01	0.85	2.38E-01	-1.22
	Phosphatidylcholines	Phosphatidylcholine (C18:0,C22:6)	1.05	1.04	1.95E-02	7.76E-02	2.44	1.81	1.02E-01	3.00E-01	0.93	5.96E-01	-0.54
	Sphingomyelins	Sphingomyelin (d18:1,C24:0)	1.00	1.07	8.63E-01	1.51E-02	0.17	2.55	9.52E-01	7.89E-02	1.04	6.42E-01	0.47
	Triacylglycerols	TAG (C16:0,C16:1)	0.88	1.00	1.13E-02	9.94E-01	-2.66	-0.01	6.22E-02	9.95E-01	0.76	5.97E-01	-0.54
	Triacylglycerols	TAG (C16:0,C18:1,C18:2)	0.82	0.94	9.97E-05	2.10E-01	-4.35	-1.28	8.01E-04	5.43E-01	1.01	9.83E-01	0.02
	Triacylglycerols	TAG (C16:0,C18:1,C18:3)<br>(add. TAG (C16:0,C18:2,C18:2),<br>TAG (C16:1,C18:1,C18:2))	0.90	1.01	2.61E-02	8.52E-01	-2.32	0.19	1.33E-01	9.60E-01	1.03	9.34E-01	0.08
	Triacylglycerols	TAG (C16:0,C18:2)	0.91	1.02	2.91E-02	6.49E-01	-2.27	0.46	1.46E-01	8.75E-01	0.85	6.59E-01	-0.45
	Triacylglycerols	TAG (C18:1,C18:2)	0.85	1.00	1.78E-04	9.64E-01	-4.15	0.05	1.25E-03	9.95E-01	1.03	9.43E-01	0.07
Energy metabolism and related	Citrate cycle	Citrate	1.05	1.08	2.00E-01	3.75E-02	1.31	2.16	5.83E-01	1.62E-01	0.94	6.43E-01	-0.47
	Citrate cycle	Isocitrate	1.12	1.20	1.33E-04	3.76E-08	4.25	6.86	1.03E-03	3.52E-07	1.01	9.58E-01	0.05
	Citrate cycle	Malate	1.06	1.54	2.23E-01	3.12E-11	1.24	9.22	5.89E-01	3.69E-10	1.20	6.62E-02	1.97
	Glycolysis/ Gluconeogenesis	Glucose-6-phosphate (4 additionals)	0.66	1.77	1.74E-04	1.07E-06	-4.17	5.83	1.25E-03	8.58E-06	1.04	8.38E-01	0.21
	Glycolysis/ Gluconeogenesis	Glycerate	1.42	1.55	7.62E-11	1.13E-13	8.91	11.26	1.07E-09	2.12E-12	1.04	5.74E-01	0.57

	Glycolysis/ Gluconeogenesis	Lactate	1.01	1.88	8.60E-01	4.05E-20	0.18	17.91	9.52E-01	1.82E-18	1.33	1.04E-01	1.72
	Glycolysis/ Gluconeogenesis	Pyruvate (add. Phosphoenolpyruvate)	0.96	0.84	2.22E-01	7.84E-07	-1.24	-5.90	5.89E-01	6.78E-06	1.14	5.16E-01	0.66
	Ketone bodies	3-Hydroxybutyrate	0.98	0.96	2.54E-01	2.75E-02	-1.16	-2.29	6.01E-01	1.26E-01	1.12	8.70E-01	0.17
Nucleobases and related	Purine metabolism	Hypoxanthine	1.82	2.59	1.14E-11	8.05E-18	9.57	15.29	1.80E-10	2.27E-16	1.13	3.19E-01	1.03
	Purine metabolism	Uric acid	1.04	1.04	7.45E-03	1.12E-02	2.83	2.67	4.19E-02	5.99E-02	1.44	1.19E-02	2.84
	Purine metabolism	Xanthine	2.12	1.98	4.82E-13	6.12E-12	10.72	9.79	9.86E-12	8.61E-11	1.16	2.72E-01	1.14
	Pyrimidine metabolism	Uridine	0.98	1.31	5.90E-01	1.37E-11	-0.54	9.60	8.22E-01	1.72E-10	0.99	9.32E-01	-0.09
Vitamins, cofactors and related	Ascorbic acid and related	Threonic acid	2.26	2.31	1.07E-14	5.35E-15	12.18	12.47	2.42E-13	1.09E-13	1.21	2.59E-01	1.17

Table S2: Metabolites in human serum that were found to be robust to pre-analytical confounders. Forty analytes with final chemical structure identity pending are not given. Statistical analysis was done via ANOVA models (see Material and Methods). A p-value of > 0.05 or a FDR of > 0.2 was considered as statistically not significant.

Ontology 1	Ontology 2	Metabolite	Serum storage 24 h, ratio versus control	Blood incubation 6 h, ratio versus control	Serum storage 24 h, p-value versus control	Blood incubation 6 h, p-value versus control	Serum storage 24 h, t-value versus control	Blood incubation 6 h, t-value versus control	Serum storage 24 h, FDR	Blood incubation 6 h, FDR	Sex, ratio male versus female	Sex, p-value male versus female	Sex, t-value male versus female
Amino acids	Amino acids, aromatic	Tryptophan	1.01	1.00	5.18E-01	8.05E-01	0.65	0.25	0.78	0.96	0.95	6.79E-01	-0.42
	Amino acids, basic	Glutamine	1.01	1.03	5.09E-01	1.18E-01	0.67	1.60	0.78	0.38	1.21	3.44E-02	2.31

Amino acids related	Amino acid metabolites	3-Hydroxyisobutyrate	1.05	1.05	2.08E-01	2.09E-01	1.28	1.28	0.59	0.54	1.27	2.62E-01	1.16
	Amino acid metabolites	beta-Aminoisobutyrate	1.03	0.96	3.79E-01	2.12E-01	0.89	-1.27	0.66	0.54	0.98	9.48E-01	-0.07
	Amino acid metabolites	Ketoleucine	1.01	0.98	7.15E-01	3.37E-01	0.37	-0.97	0.89	0.67	1.29	8.24E-04	4.11
	Creatine metabolism	Creatine	1.06	1.02	1.02E-01	5.62E-01	1.68	0.59	0.37	0.84	0.79	3.73E-01	-0.92
	Creatine metabolism	Creatinine	1.03	0.97	4.95E-01	4.70E-01	0.69	-0.73	0.77	0.79	1.26	1.01E-01	1.74
	Tryptophan metabolism	3-Indoxylsulfate	0.99	0.93	9.49E-01	5.73E-01	-0.06	-0.57	0.97	0.85	0.86	7.51E-01	-0.32
	Tryptophan metabolism	Indole-3-acetic acid	1.00	1.01	9.29E-01	8.08E-01	0.09	0.25	0.96	0.96	1.23	3.45E-01	0.97
	Tryptophan metabolism	Indole-3-lactic acid	1.02	0.99	5.49E-01	7.26E-01	0.60	-0.35	0.80	0.91	0.91	4.36E-01	-0.80
	Tyrosine metabolism	Cresol sulfate (add. m-Cresol sulfate, o-Cresol sulfate)	0.93	0.97	6.04E-01	8.42E-01	-0.52	-0.20	0.82	0.96	0.34	1.75E-02	-2.65
	Urea cycle and related	Citrulline	1.02	0.96	3.55E-01	1.01E-01	0.94	-1.68	0.65	0.35	1.26	1.55E-01	1.49
Carbohydrates and related	Aminosugars	Mannosamine	0.96	0.88	6.12E-01	8.08E-02	-0.51	-1.80	0.82	0.30	1.15	1.44E-01	1.54
	Polyols	1,5-Anhydrosorbitol	0.98	0.97	1.64E-01	8.69E-02	-1.42	-1.76	0.51	0.32	1.52	2.04E-02	2.57
	Polyols	Erythrol	1.01	1.05	7.72E-01	1.29E-01	0.29	1.55	0.93	0.40	0.96	7.02E-01	-0.39
Complex lipids, fatty acids and related	Alkane hydrocarbons	Hentriacontane	0.99	0.99	6.49E-01	8.01E-01	-0.46	-0.25	0.84	0.96	0.73	7.33E-02	-1.92
	Bile acids	Glycocholic acid	0.92	0.89	6.03E-01	4.87E-01	-0.53	-0.70	0.82	0.79	0.94	9.37E-01	-0.08
	Bile acids	Taurocholic acid	0.94	0.96	6.10E-01	6.95E-01	-0.51	-0.39	0.82	0.90	0.40	2.17E-01	-1.29

	Ceramides	Ceramide (d18:1,C24:0)	1.07	1.02	1.14E-01	6.55E-01	1.62	0.45	0.38	0.88	0.85	4.79E-01	-0.73
	Cholesterol and related	Cholesta-2,4,6-triene	1.04	0.94	5.66E-01	3.96E-01	0.58	-0.86	0.81	0.75	0.77	1.62E-01	-1.47
	Cholesterol and related	Cholesta-2,4-dien	0.97	0.99	7.10E-01	8.93E-01	-0.37	-0.14	0.89	0.97	0.66	5.15E-02	-2.10
	Cholesterol and related	Cholestenol No 02	1.05	1.02	3.91E-01	7.22E-01	0.87	0.36	0.66	0.91	0.73	9.94E-02	-1.75
	Cholesterol and related	Cholesterol, free	0.92	1.00	2.28E-01	9.95E-01	-1.23	-0.01	0.59	1.00	0.91	2.23E-01	-1.27
	Cholesterol and related	Cholesterol, total	0.97	0.98	2.39E-01	4.43E-01	-1.20	-0.78	0.59	0.79	0.74	4.83E-02	-2.14
	Cholesterylesters	Cholesterylester C20:4	1.20	1.11	5.24E-02	2.82E-01	2.00	1.09	0.23	0.64	0.81	4.83E-01	-0.72
	Diacylglycerols	DAG (C18:1,C18:2)	1.14	1.04	2.27E-01	6.77E-01	1.23	0.42	0.59	0.89	1.04	8.96E-01	0.13
	Fatty acids, branched	14-Methylhexadecanoic acid	1.01	1.02	6.41E-01	4.35E-01	0.47	0.79	0.84	0.79	0.59	1.17E-01	-1.66
	Fatty acids, mono-unsaturated	Elaidic acid (C18:trans[9]1)	0.96	0.97	3.46E-01	4.71E-01	-0.96	-0.73	0.65	0.79	0.83	4.15E-01	-0.84
	Fatty acids, mono-unsaturated	Nervonic acid (C24:cis[15]1)	1.05	1.03	6.89E-02	3.01E-01	1.88	1.05	0.28	0.64	0.69	4.28E-02	-2.20
	Fatty acids, mono-unsaturated	Oleic acid (C18:cis[9]1)	1.01	1.02	6.47E-01	4.67E-01	0.46	0.74	0.84	0.79	0.81	4.76E-01	-0.73
	Fatty acids, mono-unsaturated	Palmitoleic acid (C16:cis[9]1)	0.95	0.99	1.08E-01	8.64E-01	-1.65	-0.17	0.38	0.96	0.62	2.97E-01	-1.08
	Fatty acids, poly-unsaturated	Arachidonic acid (C20:cis[5,8,11,14]4)	1.00	1.01	8.19E-01	6.01E-01	-0.23	0.53	0.95	0.86	0.91	6.67E-01	-0.44
	Fatty acids, poly-unsaturated	Conjugated Linoleic acid (C18:trans[9,11]2) (add. conjugated Linoleic acid (C18:cis[9]trans[11]2))	0.97	1.03	3.68E-01	4.87E-01	-0.91	0.70	0.65	0.79	0.79	3.45E-01	-0.97
	Fatty acids, poly-unsaturated	dihomo-gamma-Linolenic acid (C20:cis[8,11,14]3)	1.02	1.02	3.20E-01	3.44E-01	1.01	0.96	0.63	0.68	0.76	3.53E-01	-0.96

	Fatty acids, poly-unsaturated	Docosapentaenoic acid (C22:cis[4,7,10,13,16]5)	0.98	1.01	6.51E-01	8.60E-01	-0.46	0.18	0.84	0.96	0.62	1.72E-01	-1.44
	Fatty acids, poly-unsaturated	Docosapentaenoic acid (C22:cis[7,10,13,16,19]5)	0.96	0.99	3.10E-01	8.54E-01	-1.04	-0.19	0.63	0.96	1.56	6.84E-02	2.02
	Fatty acids, poly-unsaturated	Eicosadienoic acid (C20:2) No 02	0.98	1.02	7.94E-01	8.22E-01	-0.26	0.23	0.95	0.96	0.79	4.40E-01	-0.79
	Fatty acids, poly-unsaturated	Eicosapentaenoic acid (C20:cis[5,8,11,14,17]5)	1.02	1.03	4.36E-01	2.62E-01	0.79	1.15	0.70	0.63	1.28	4.52E-01	0.78
	Fatty acids, poly-unsaturated	gamma-Linolenic acid (C18:cis[6,9,12]3)	1.03	1.01	3.64E-01	8.02E-01	0.92	0.25	0.65	0.96	0.89	7.54E-01	-0.32
	Fatty acids, poly-unsaturated	Linoleic acid (C18:cis[9,12]2)	1.00	1.01	8.66E-01	5.59E-01	0.17	0.59	0.95	0.84	0.72	1.38E-01	-1.56
	Fatty acids, saturated	Behenic acid (C22:0)	0.98	1.00	4.03E-01	9.79E-01	-0.85	0.03	0.67	1.00	0.76	1.63E-02	-2.68
	Fatty acids, saturated	Eicosanoic acid (C20:0)	0.96	1.00	1.23E-01	9.91E-01	-1.58	-0.01	0.40	1.00	0.67	7.11E-04	-4.18
	Fatty acids, saturated	Heptadecanoic acid (C17:0)	0.94	0.96	5.65E-02	2.43E-01	-1.97	-1.19	0.24	0.60	0.77	2.06E-01	-1.32
	Fatty acids, saturated	Lignoceric acid (C24:0)	0.98	0.99	3.73E-01	6.31E-01	-0.90	-0.48	0.65	0.87	0.83	2.26E-01	-1.26
	Fatty acids, saturated	Palmitic acid (C16:0)	0.99	1.01	8.01E-01	6.16E-01	-0.25	0.51	0.95	0.86	0.81	4.53E-01	-0.77
	Fatty acids, saturated	Stearic acid (C18:0)	0.99	1.01	7.36E-01	5.92E-01	-0.34	0.54	0.90	0.86	0.84	3.39E-01	-0.98
	Fatty acids, saturated	Tricosanoic acid (C23:0)	1.02	1.04	6.27E-01	2.69E-01	0.49	1.12	0.84	0.63	0.61	1.07E-02	-2.89
	Fatty alcohols	Dodecanol	0.91	0.93	1.83E-01	3.06E-01	-1.36	-1.04	0.55	0.64	0.86	4.49E-01	-0.78
	Fatty alcohols	Glycerol, lipid fraction	1.00	1.02	9.31E-01	5.40E-01	0.09	0.62	0.96	0.82	0.90	8.09E-01	-0.25
	Fatty alcohols	Hexadecanol	0.98	1.00	6.39E-01	9.11E-01	-0.47	-0.11	0.84	0.98	0.93	3.45E-01	-0.97

	Fatty alcohols	Pentadecanol	0.98	1.02	5.24E-01	6.34E-01	-0.64	0.48	0.78	0.87	0.92	8.19E-01	-0.23
	Fatty alcohols	Tetradecanol	0.94	0.97	2.03E-01	4.59E-01	-1.30	-0.75	0.59	0.79	0.95	4.03E-01	-0.86
	Glycolipids	Galactose, lipid fraction	1.00	1.01	9.70E-01	8.99E-01	-0.04	0.13	0.98	0.97	0.86	9.45E-02	-1.78
	Glycolipids	Glucose, lipid fraction	0.99	0.96	9.39E-01	7.88E-01	-0.08	-0.27	0.96	0.96	1.23	1.52E-01	1.51
	Lipid hydroperoxides	Cholesterylester hydroperoxide (C18:2-9-OOH) (add. Cholesterylester hydroperoxide (C18:2-13-OOH))	1.29	1.16	2.42E-01	4.86E-01	1.19	0.71	0.59	0.79	1.11	6.45E-01	0.47
	Lipid hydroperoxides	Phosphatidylcholine hydroperoxide (C16:0,C18:1-OOH)	0.74	0.69	1.13E-01	6.51E-02	-1.63	-1.90	0.38	0.26	1.17	6.85E-01	0.41
	Lipid hydroperoxides	Phosphatidylcholine hydroperoxide (C18:0,C18:2-OOH)	0.95	0.98	5.24E-01	8.45E-01	-0.64	-0.20	0.78	0.96	0.99	9.65E-01	-0.04
	Lipid hydroperoxides	Triacylgyceride hydroperoxide (C16:0,C18:1,C18:2-OOH)	0.96	1.02	8.40E-01	9.32E-01	-0.20	0.09	0.95	0.99	0.75	2.52E-01	-1.19
	Lipid hydroperoxides	Triacylgyceride hydroperoxide (C16:0,C18:1,C18:3-OOH) (add. Triacylgyceride hydroperoxide (C16:0,C18:2,C18:2-OOH))	0.96	1.14	8.37E-01	4.58E-01	-0.21	0.75	0.95	0.79	0.58	8.88E-02	-1.81
	Lipid hydroperoxides	Triacylgyceride hydroperoxide (C18:1,18:2,C18:2-OOH) (add. Triacylgyceride hydroperoxide (C16:0,C18:1,C20:4-OOH), Triacylgyceride hydroperoxide (C18:1,C18:1,C18:3-OOH))	0.83	1.00	3.08E-01	9.89E-01	-1.03	-0.01	0.63	1.00	0.77	3.73E-01	-0.92
	Lipid precursors	Glycerol, polar fraction	1.17	1.17	8.55E-02	9.04E-02	1.77	1.74	0.34	0.33	0.89	5.89E-01	-0.55
	Lysophosphatidylcholines	Lysophosphatidylcholine (C18:2)	1.09	1.03	5.13E-02	5.58E-01	2.01	0.59	0.23	0.84	1.18	4.67E-01	0.75
	Lysophosphatidylethanolamines	Lysophosphatidylethanolamine (C22:5)	0.93	0.92	2.68E-01	1.79E-01	-1.12	-1.37	0.61	0.52	0.92	5.88E-01	-0.55
	Phosphatidylcholines	Phosphatidylcholine (C16:0,C16:0)	0.97	0.98	2.53E-01	3.23E-01	-1.16	-1.00	0.60	0.66	0.90	1.21E-01	-1.64
	Phosphatidylcholines	Phosphatidylcholine (C16:0,C18:2)	1.01	1.01	8.61E-02	5.00E-01	1.76	0.68	0.34	0.79	1.02	1.83E-01	1.39

	Phosphatidylcholines	Phosphatidylcholine (C16:0,C20:4) (add. Phosphatidylcholine (C18:2,C18:2))	0.99	1.00	2.45E-01	4.04E-01	-1.18	-0.84	0.59	0.76	1.01	4.82E-01	0.72
	Phosphatidylcholines	Phosphatidylcholine (C16:0,C20:5)	0.96	1.00	1.53E-01	9.63E-01	-1.46	0.05	0.48	1.00	0.99	9.38E-01	-0.08
	Phosphatidylcholines	Phosphatidylcholine (C16:0,C22:6) (add. Phosphatidylcholine (C18:2,C20:4))	1.01	0.99	5.64E-01	3.94E-01	0.58	-0.86	0.81	0.75	0.89	1.92E-01	-1.36
	Phosphatidylcholines	Phosphatidylcholine (C18:0,C18:1)	0.99	1.01	1.94E-01	1.84E-01	-1.32	1.35	0.58	0.52	0.87	2.34E-02	-2.50
	Phosphatidylcholines	Phosphatidylcholine (C18:0,C18:2)	1.02	1.01	1.44E-01	2.11E-01	1.49	1.27	0.46	0.54	1.00	8.73E-01	-0.16
	Phosphatidylcholines	Phosphatidylcholine (C18:0,C20:3) (add. Phosphatidylcholine (C20:1,C18:2), Phosphatidylcholine (C20:2,C18:1))	1.00	1.00	9.41E-01	6.47E-01	0.07	0.46	0.96	0.87	0.96	5.86E-01	-0.56
	Phosphatidylcholines	Phosphatidylcholine (C18:0,C20:4)	1.00	0.99	8.49E-01	1.95E-01	0.19	-1.32	0.95	0.54	1.01	8.36E-01	0.21
	Phosphatidylcholines	Phosphatidylcholine (C18:1,C18:2) (add. Phosphatidylcholine (C16:0,C20:3))	1.01	1.00	9.12E-02	6.02E-01	1.73	0.53	0.35	0.86	0.98	6.60E-02	-1.97
	Phosphatidylcholines	Phosphatidylcholine No 02	1.02	1.01	3.17E-01	7.72E-01	1.01	0.29	0.63	0.95	1.02	7.86E-01	0.28
	Phospholipid metabolites	Glycerol phosphate, lipid fraction	0.99	0.97	8.23E-01	5.94E-01	-0.23	-0.54	0.95	0.86	0.66	7.18E-02	-1.93
	Phospholipid metabolites	myo-Inositol-2-phosphate, lipid fraction (myo-Inositolphospholipids)	0.89	0.93	2.96E-01	5.26E-01	-1.06	-0.64	0.63	0.80	0.58	9.62E-02	-1.77
	Phospholipid metabolites	Phosphate, lipid fraction	1.00	1.02	9.31E-01	2.40E-01	-0.09	1.20	0.96	0.60	0.76	3.57E-02	-2.29
	Plasmalogens	Choline plasmalogen (C18,C20:4)	1.03	0.97	2.82E-01	2.43E-01	1.09	-1.19	0.63	0.60	1.04	6.99E-01	0.39
	Sphingolipids	1-Hydroxy-2-amino-(cis,trans)-3,5-octadecadiene (from sphingolipids)	1.03	1.02	3.01E-01	4.93E-01	1.05	0.69	0.63	0.79	0.65	5.81E-03	-3.18
	Sphingolipids	3-O-Methylsphingosine (d18:1) (add. Sphingolipids, erythro-Sphingosine (d18:1), threo-Sphingosine (d18:1))	1.02	1.02	4.33E-01	5.13E-01	0.79	0.66	0.70	0.79	0.65	5.03E-03	-3.25

	Sphingolipids	4-Hydroxysphinganine (t18:0, Phytosphingosine), total	0.98	0.97	6.38E-01	5.87E-01	-0.47	-0.55	0.84	0.86	0.74	4.79E-02	-2.14
	Sphingolipids	5-O-Methylsphingosine (d18:1) (add. Sphingolipids, erythro-Sphingosine (d18:1), threo-Sphingosine (d18:1))	1.02	1.02	3.96E-01	3.27E-01	0.86	0.99	0.66	0.66	0.68	9.86E-03	-2.93
	Sphingolipids	erythro-Dihydrosphingosine (d16:0)	1.00	1.00	9.26E-01	8.55E-01	0.09	0.18	0.96	0.96	0.72	6.23E-02	-2.00
	Sphingolipids	erythro-Dihydrosphingosine (d18:0)	1.02	1.00	3.20E-01	8.66E-01	1.01	0.17	0.63	0.96	0.76	2.03E-01	-1.33
	Sphingolipids	erythro-Sphingosine (d18:1) (add. Sphingolipids)	1.01	1.01	7.07E-01	7.09E-01	0.38	0.38	0.89	0.91	0.69	3.65E-03	-3.40
	Sphingolipids	threo-Sphingosine (d18:1) (add. Sphingolipids)	1.00	1.01	9.28E-01	6.63E-01	0.09	0.44	0.96	0.88	0.72	8.80E-03	-2.98
	Sphingomyelins	Sphingomyelin (d18:1,C16:0)	0.99	0.97	5.78E-01	6.39E-02	-0.56	-1.91	0.82	0.26	1.01	4.82E-01	0.72
	Sphingomyelins	Sphingomyelin (d18:1,C23:0)	0.95	0.97	2.31E-01	5.15E-01	-1.22	-0.66	0.59	0.79	0.76	2.69E-02	-2.44
	Sphingomyelins	Sphingomyelin (d18:1,C24:1) (add. Sphingomyelin (d18:2,C24:0))	0.94	0.97	7.92E-01	9.09E-01	-0.26	-0.11	0.95	0.98	0.99	9.57E-01	-0.05
	Sphingomyelins	Sphingomyelin (d18:2,C18:0)	1.01	1.00	3.70E-01	8.19E-01	0.91	-0.23	0.65	0.96	0.75	1.04E-03	-4.00
	Sphingomyelins	Sphingomyelin No 01	1.02	1.05	5.33E-01	2.04E-01	0.63	1.29	0.78	0.54	0.88	2.13E-01	-1.30
	Triacylglycerols	TAG (C18:1,C18:2,C18:3) (add. TAG (C16:0,C18:1,C20:5), TAG (C16:0,C18:2,C20:4))	0.96	0.93	3.24E-01	9.30E-02	-1.00	-1.72	0.63	0.33	1.12	5.35E-01	0.63
	Triacylglycerols	TAG (C18:2,C18:2)	1.01	1.04	8.48E-01	4.90E-01	0.19	0.70	0.95	0.79	0.96	9.04E-01	-0.12
Energy metabolism and related	Citrate cycle	alpha-Ketoglutarate	0.92	1.00	4.13E-01	9.81E-01	-0.83	-0.02	0.67	1.00	1.49	3.40E-02	2.32
	Citrate cycle	Succinate	0.97	0.98	2.61E-01	2.87E-01	-1.14	-1.08	0.61	0.64	0.97	1.68E-01	-1.45
	Energy metabolism, miscellaneous	2-Hydroxybutyrate	1.02	1.03	3.21E-01	1.80E-01	1.00	1.37	0.63	0.52	1.29	3.96E-01	0.87

Hormones, signal substances and related	Steroids and related	Cortisol	0.77	0.82	4.38E-02	1.10E-01	-2.09	-1.63	0.20	0.36	0.57	1.20E-01	-1.64
	Steroids and related	Dehydroepiandrosterone sulfate (add. Testosterone-17-sulfate)	0.93	0.89	4.53E-01	2.02E-01	-0.76	-1.30	0.72	0.54	1.33	4.29E-01	0.81
Miscellaneous	Diet related	beta-Sitosterol	1.02	1.07	7.38E-01	3.06E-01	0.34	1.04	0.90	0.64	1.12	5.48E-01	0.61
	Diet related	Canthaxanthin	0.89	0.96	1.84E-01	6.07E-01	-1.35	-0.52	0.55	0.86	0.72	5.01E-01	-0.69
	Diet related	Cryptoxanthin	1.05	1.00	5.92E-01	9.95E-01	0.54	-0.01	0.82	1.00	0.68	3.05E-01	-1.06
	Diet related	Quinic acid (add. Chlorogenic acid (CGA))	1.06	0.91	3.27E-01	1.81E-01	1.00	-1.37	0.63	0.52	0.35	2.16E-01	-1.29
	Miscellaneous	4-Deoxythreonic acid	1.00	0.99	9.98E-01	8.77E-01	0.00	-0.16	1.00	0.96	1.64	1.12E-01	1.68
	Miscellaneous	Benzoic acid	1.12	0.90	3.94E-01	4.56E-01	0.86	-0.75	0.66	0.79	1.32	3.06E-01	1.06
	Miscellaneous	Glycolate	1.04	1.00	4.91E-01	9.46E-01	0.70	-0.07	0.77	1.00	1.02	7.87E-01	0.27
	Miscellaneous	Hippuric acid	0.98	0.96	7.57E-01	5.02E-01	-0.31	-0.68	0.92	0.79	0.59	1.72E-01	-1.43
	Miscellaneous	Phosphate (inorganic and from organic phosphates)	1.02	1.00	3.03E-01	9.60E-01	1.04	-0.05	0.63	1.00	1.02	8.40E-01	0.21
Nucleobases and related	Pyrimidine metabolism	Pseudouridine	1.04	1.04	3.64E-01	2.99E-01	0.92	1.05	0.65	0.64	0.99	8.97E-01	-0.13
Vitamins, cofactors and related	Acyl-carriers and related	Pantothenic acid	1.02	1.08	6.67E-01	5.76E-02	0.43	1.96	0.84	0.24	1.00	9.94E-01	-0.01
	Amino-carriers and related	4-Pyridoxic acid	0.95	0.93	2.13E-01	7.85E-02	-1.27	-1.81	0.59	0.30	0.86	6.53E-01	-0.46
	Carotenoids	beta-Carotene	1.00	0.99	9.24E-01	8.89E-01	-0.10	-0.14	0.96	0.97	0.62	1.11E-01	-1.69
	Redox-carrier and related	Coenzyme Q10	0.98	1.04	7.16E-01	5.01E-01	-0.37	0.68	0.89	0.79	1.23	3.41E-01	0.98

	Tocopherols and related	alpha-Tocopherol	1.00	1.02	9.75E-01	3.95E-01	-0.03	0.86	0.98	0.75	1.08	4.08E-01	0.85
